# Surgery for craniovertebral junction pathologies: minimally invasive anterior submandibular retropharyngeal key-hole approach

**DOI:** 10.1186/s12893-021-01198-z

**Published:** 2021-04-19

**Authors:** Árpád Viola, István Kozma, Dávid Süvegh

**Affiliations:** 1 Department of Neurotraumatology, Semmelweis University, Fiumei út 17, 1081 Budapest, Hungary; 2 Department of Neurosurgery and Neurotraumatology, Péterfy Hospital - Manninger Jenő National Traumatology Institution, Fiumei út 17, 1081 Budapest, Hungary; 3 Péterfy Hospital - Manninger Jenő National Traumatology Institution, Fiumei út 17, 1081 Budapest, Hungary

**Keywords:** Odontoidectomy, Minimally invasive, Key-hole approach, Submandibular retropharyngeal, Ventral decompression, Patient safety

## Abstract

**Background:**

Our objective was to develop a new, minimally invasive surgical technique for the resolution of craniovertebral junction pathologies, which can eliminate the complications of the previous methods, like liquor-leakage, velopharyngeal insufficiency and wound-dehiscence associated with the transoral or lateral approaches.

**Methods:**

During the first stage of the operation, three patients underwent occipito-cervical dorsal fusion, while the fourth patient received C1–C2 fusion according to Harms. C1–C2 decompressive laminectomy was performed in all four cases. Ventral C1–C2 decompression with microscope assisted minimally invasive anterior submandibular retropharyngeal key-hole approach (MIS ASR) method was performed in the second stage. The MIS ASR—similarly to the traditional anterior retropharyngeal surgery—preserves the hard and soft palates, yet can be performed through a 25 mm wide incision with the use of only one retractor.

**Results:**

The MIS ASR approach was a success in all four cases, there were no intra- and postoperative complications. This method, compared to the transoral approach, provided on average 23% (4.56 cm^2^/6.05 cm^2^) smaller dural decompression area; nonetheless, the entire pathology could be removed in all cases. After the surgery, all patients have shown significant neurological improvement.

**Conclusion:**

Based on the outcome of these four cases we think that the MIS ASR approach is a safe alternative to the traditional methods while improving patient safety by reducing the risk of complications.

**Supplementary Information:**

The online version contains supplementary material available at 10.1186/s12893-021-01198-z.

## Background

Odontoidectomy serves as the resolution of ventral compression of the upper cervical medulla. It can be performed via traditional transoral, endoscopic endonasal, anterior transcervical retropharyngeal, or with a lateral approach. The most common method for ventral decompression is by transoral transpharyngeal (TO) odontoidectomy [1] with the option to add a transmandibular route or Le Fort osteotomy for increased visualization and surgical bed [2]. The benefit of endoscopic endonasal (EE) odontoidectomy over the transoral method is that the preservation of the hard and soft palates drastically decreases the risk of velopharyngeal insufficiency, while a straight approach to the odontoid process is still provided [3–5]. The risks and limitations of transmucosal surgeries can be avoided with an anterior-transcervical-retropharyngeal approach. Using the standard Smith-Robinson method, access to the C3 vertebra and disc is not always attainable, while the anterior retropharyngeal (AR) approach provides access to the whole cervical spine [6, 7]. Although lateral routes provide wider access, the risk of injuries to the vertebral artery, jugular bulb and hypoglossal nerve are higher [8–11]. Our objective is to introduce a surgical method that we have used for C1–C2 ventral decompression, the microscope assisted minimally invasive anterior submandibular retropharyngeal key-hole approach (MIS ASR), which—similarly to the traditional anterior retropharyngeal surgery—preserves the hard and soft palates, yet can be performed through a 25 mm wide incision with the use of only one retractor.

## Methods

**Fig. 1:**
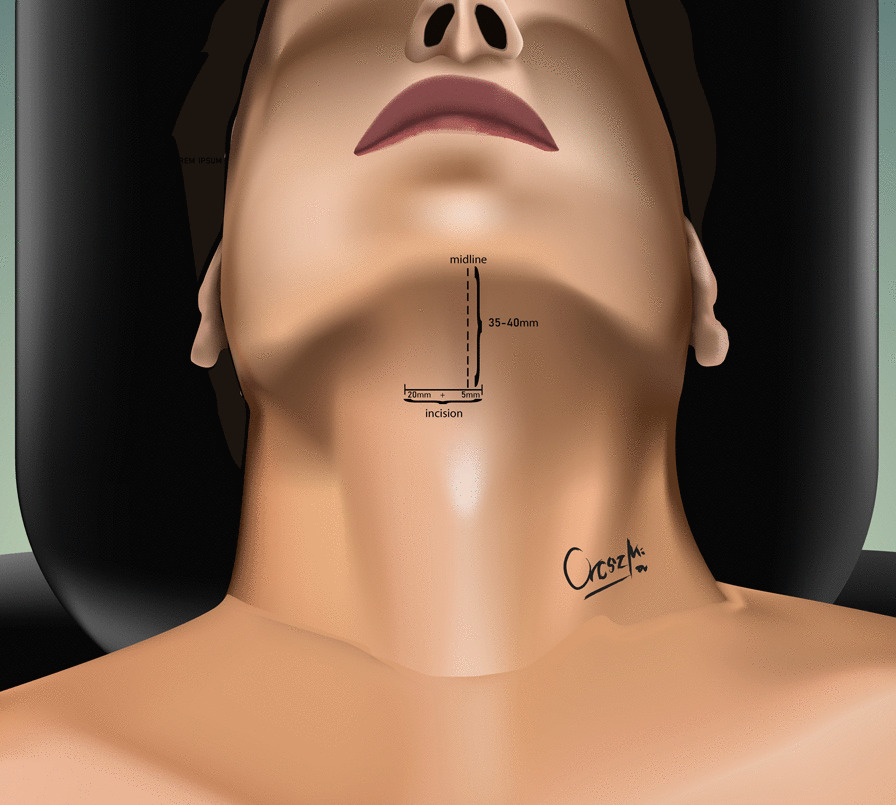
Location of skin incision

**Fig. 2:**
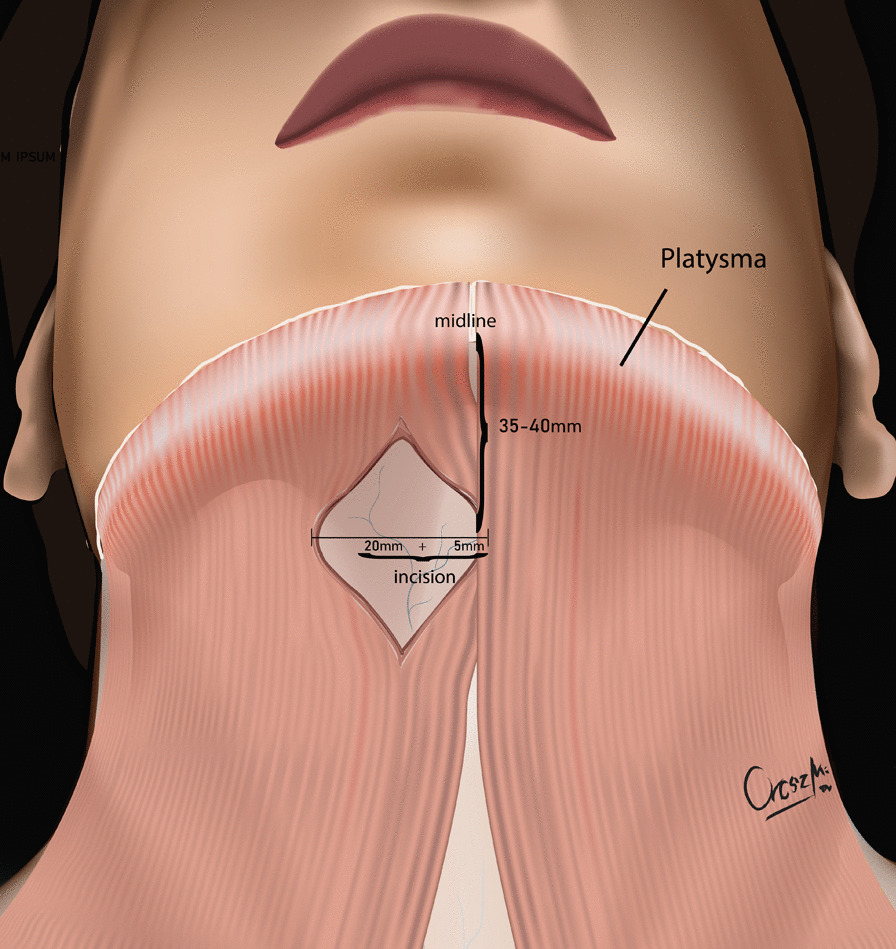
Vertical dissection of the platysma muscle

**Fig. 3:**
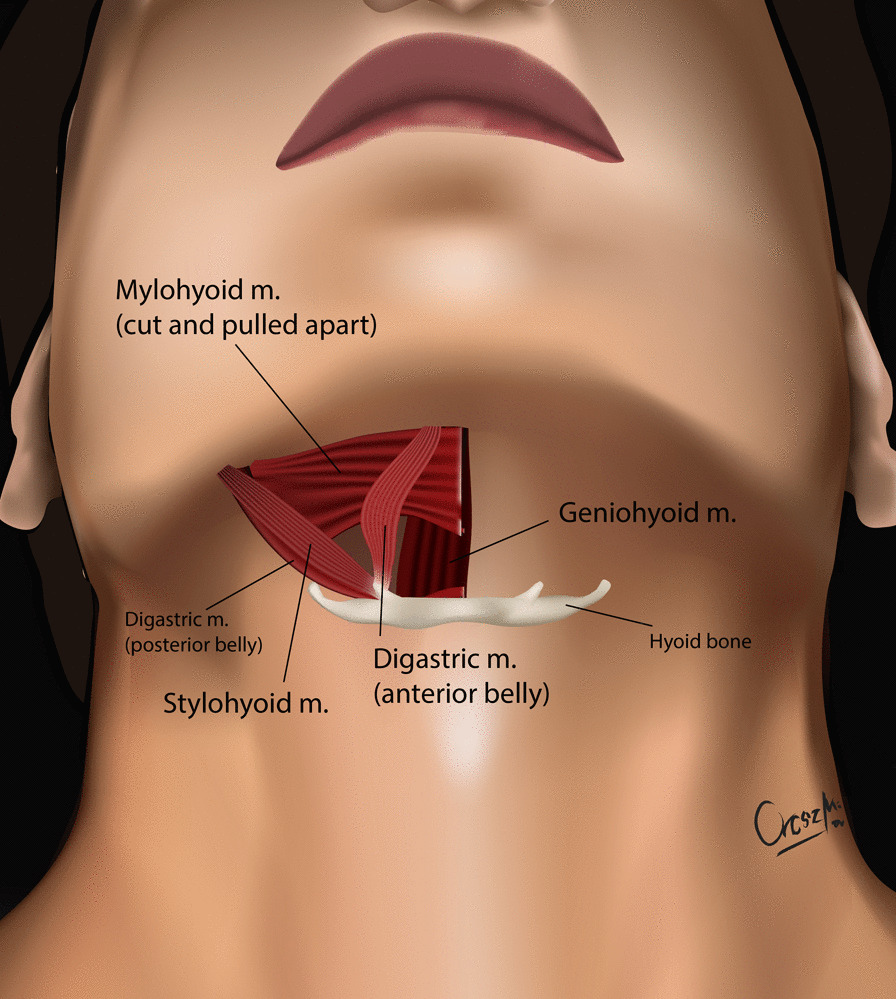
Retracting and dissecting the deeper muscles of the neck to reach the spinal column

**Fig. 4:**
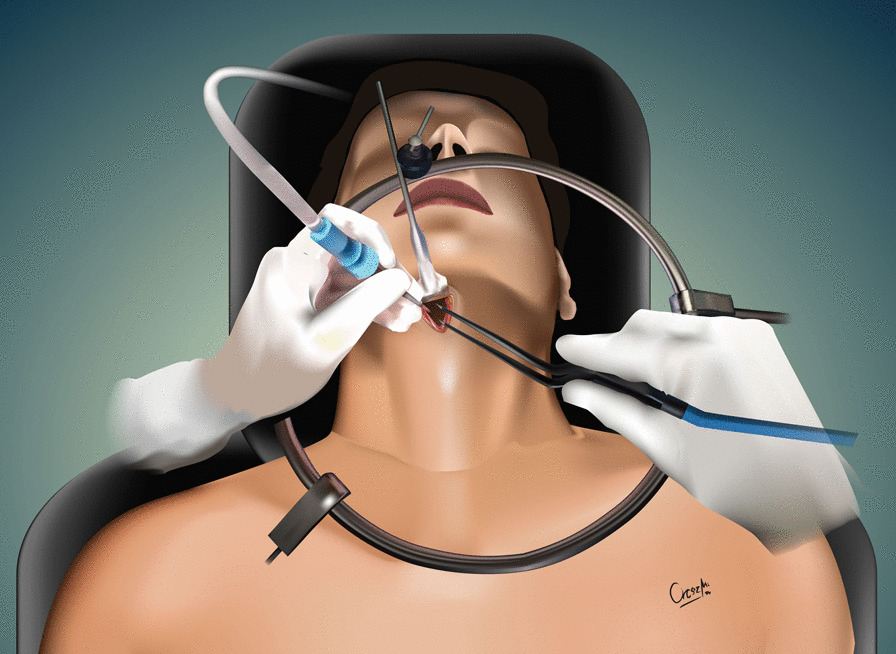
The carbon retractor attaches to an external holding ring (DePuy Synthes Synframe)

**Fig. 5:**
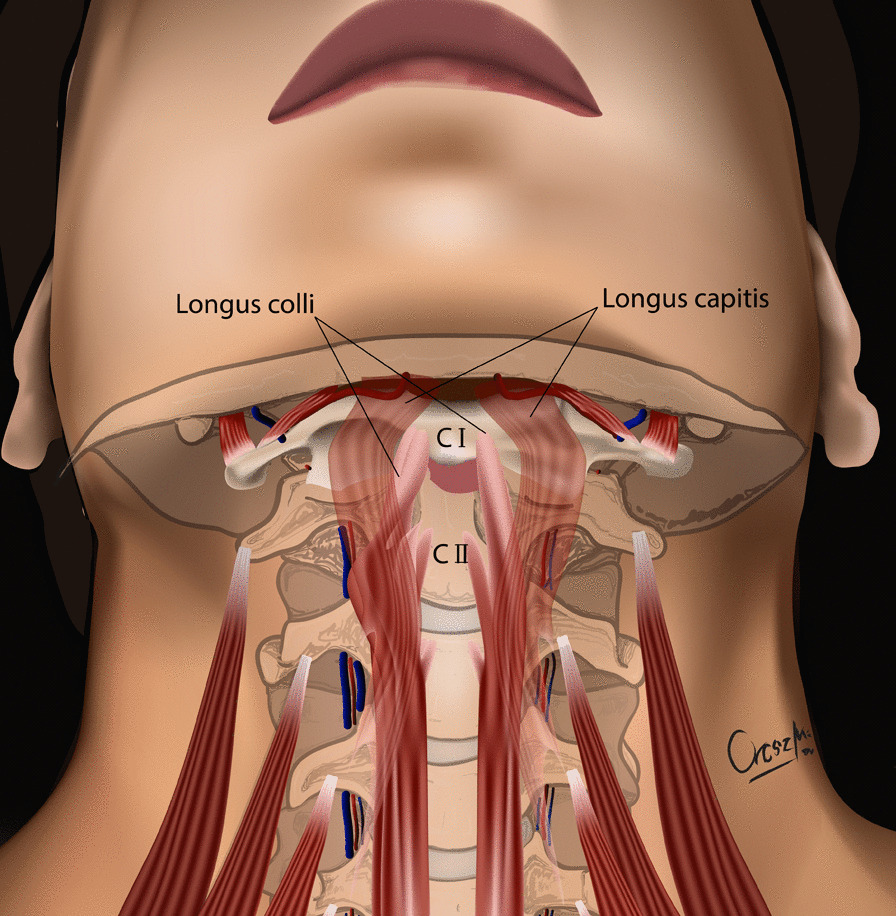
Insertion of longus colli muscle

**Fig. 6:**
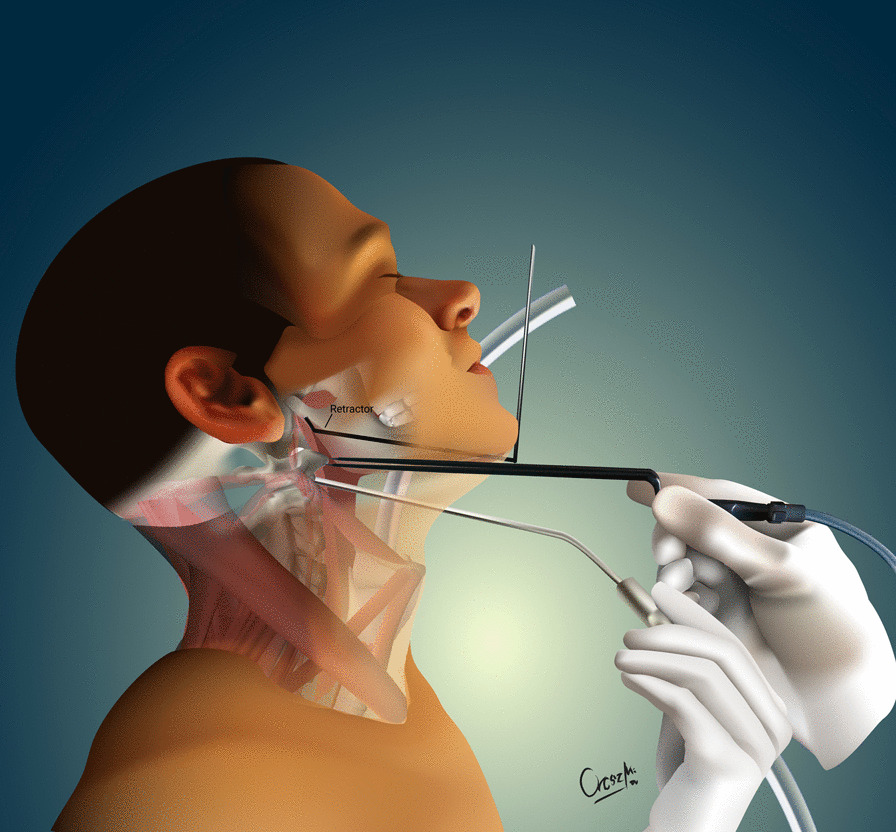
Mobilization of oropharynx with retractor and retraction of soft tissue with bipolar diathermy and suction

The development of the MIS ASR procedure was inspired by a trauma case. A cortical bone fragment broke off from the odontoid process of the C2 vertebra and compressed the spinal cord on the left side [12]. We chose the MIS ASR, because the MRI confirmed a ventral dura injury, which along with the high risk of velopharyngeal insufficiency occurring with the transoral approach, would have increased the risk of wound dehiscence and sepsis. With the submandibular “key-hole” approach, besides using fibrin glue, we could also tamponade the dural injury with multiple layers of vital soft tissue. During the surgery and in the postoperative period, no cerebrospinal fluid (CSF) leakage or other complications presented. After this successful operation on the trauma patient, we began to utilize the MIS ASR key-hole method to assess its feasibility in rheumatoid arthritis and tumorous cases, the two most common reasons for ventral compression. The surgery was performed on two patient in his 60 s and 80 s patients with ventral spinal stenosis caused by rheumatoid arthritis, on a patient in her 40 s to whom the ventral spinal stenosis was due to a metastasis of a cervix squamous-cell carcinoma, and on a patient in his 40 s with the traumatic odontoid fracture with dislocation resulting in the compression of the medulla oblongata. During the first stage of the operation, three patients underwent occipito-cervical dorsal fusion, while the fourth patient received C1–C2 fusion according to Harms. C1–C2 decompressive laminectomy was performed in all four cases. Ventral C1–C2 decompression was performed in the second stage. All patients were supine, stabilized in Mayfield headrest. During the MIS ASR approach, we began with a 25 mm wide ventral and submandibular incision, 30–40 mm below the mental protuberance but cranially from the hyoid bone, beginning from the right side and extending 5 mm left towards the midline (Fig. 1). After the skin incision, we visualized the cervical fascia, which we opened with surgical scissors. The platysma muscle was bluntly dissected vertically (Fig. 2), then we retracted laterally the anterior belly of the right digastric muscle. This unfolds the mylohyoid muscle, which we dissected bluntly, horizontally, corresponding to its fibers (Fig. 3). Below that, the geniohyoid muscle was dissected vertically between its fibers. At this point, we inserted a 15 mm wide–80 mm long carbon retractor (DePuy Synthes Synframe) reaching the ventral surface of C1 and C2 and secured it to the external holding ring (Fig. 4). With this retractor in place, we mobilized the upper portion of the oropharynx cranio-medially to give us access to the upper cervical spine. We could then mobilize the insertion of the longus colli muscle from the anterior tubercle of the atlas (Fig. 5), to freely resect the C1 vertebra’s anterior arch, the odontoid process and the upper portion of the C2 vertebra. According to the different stages of the surgery, the suction, bipolar diathermy, micro drill and Kerrison Rongeur were positioned in the surgeon’s left and right hand in order to provide adequate retraction of the lateral and medial soft tissue (Fig. 6).

## Results

Using this microscope assisted minimally invasive anterior submandibular retropharyngeal key-hole (MIS ASR) approach, C1–C2 ventral decompression and freeing up of the dura mater can be performed. Operative time in the four cases were 165, 150, 135 and 130 min respectively—145 min on average, and the blood loss was between 80 and 120 ml. Tracheostomy was not needed in any of the cases. The patients were extubated immediately after the procedure. They spent 12 h in the intensive care unit for observation and were started on an oral diet within 24 h. There were no intra- or postoperative complications, and significant neurological improvement presented in every case. An additional movie shows the surgery of the rheumatoid arthritis patient in his 60 s in detail (Additional file 1). The mean follow-up time was 8.25 months (15, 8, 4, 6). The 83-year-old male operated due to the rheumatoid pannus died four months after the surgery in Clostridium difficile sepsis. The other three patients are alive, able to walk and are self-sufficient. During the four surgeries—on average—we operated 94.3 mm deep (108, 95.7, 90.3 and 83 mm), measured from the skin incision. This was calculated by averaging the distance measured from the skin surface to the C1 tubercle, the ventral surface of the C2 and the deepest point of the freed-up dura in all four cases (altogether 12 values). In each patient, the anterior tubercle of the C1 vertebra was 105, 98, 95, 78 mm deep, while the ventral base of the C2 was 91, 79, 74, 70 mm respectively. After the removal of bone and soft tissue, the dura mater was 128, 110, 102, 101 mm deep from the incision (Table 1). The area of decompression was 5.44, 4.84, 4.83 and 3.50–4.65 cm^2^ in average. This area was measured as a rectangle on the post-operative CT scans, in the coronal plane, in the level of the ventral surface of the dura. We planned the surgeries with the TO method using CT scans. In these cases, the mean length of the surgical channel would have been 89.8 mm (86.3, 98.7, 90.3 and 83.7 mm)–4.5 mm less than with the MIS ASR. The maximum possible area of decompression, measured in the coronal plane on the CT scans, would have been 7.35, 5.81, 5.61 and 5.44 cm^2^ to 6.05 cm^2^ in average.

**Table 1:** The length of the surgical channel with the MIS ASR and the TO method

No. of patient	MIS ASR				TO			
	I-C1 (mm)	I-C2 (mm)	I-D (mm)	DF (cm^2^)	O-C1 (mm)	O-C2 (mm)	O-D (mm)	DF (cm^2^)
1	105	91	128	3.50	76	85	98	5.81
2	98	79	110	4.83	89	96	111	7.35
3	95	74	102	5.44	77	93	101	5.44
4	78	70	101	4.84	74	86	91	5.61

*MIS ASR* minimally invasive anterior submandibular retropharyngeal key-hole approach, *TO* transoral approach, *I-C1* distance between the incision and C1 vertebra’s anterior tubercle, *I-C2* distance between the incision and the ventral base of C2 vertebra, *I-D* distance between the incision and the deepest point of the dura mater, *DF* decompressed dura mater surface, *O-C1* distance between the orifice and C1 vertebra’s anterior tubercle, *O-C2* distance between the orifice and the ventral base of C2 vertebra, *O-D* distance between the orifice and the deepest point of the dura mater

### Patient one

We admitted male in his 80 s from a neurological department, with 2-month persistent symptoms and slowly developing paraparesis. The MRI identified a rheumatoid pannus causing ventral compression at the C1–2 level. Two days after the posterior C1–2 fixation and laminectomy, we performed the MIS ASR surgery without any complications. After the procedure, the patient’s paraparesis gradually resolved itself and he became self-sufficient.

### Patient two

The male in his 60 s presented with moderate tetraparesis, dysphagia, and Bechterew disease in the anamnesis. From the MRI scans we identified a rheumatoid pannus compressing the medulla oblongata. Nine days after the dorsal C1–2 fusion we performed the MIS ASR surgery, during which the anterior arch of C1, the upper two-thirds of the odontoid process, and the pannus causing the compression were removed (Additional file 1). The surgery was completed without complications. The neurological deficit, dysphagia and tetraparesis gradually resolved themselves, and 10 days after the second surgery the patient was discharged to his home.

### Patient three

With the female in her 40 s, the CT and contrast MRI showed the C1 and C2 vertebra’s tumorous infiltration—due to a metastatic cervix squamous cell carcinoma metastasis—which caused the ventral compression of the spinal cord. She had no neurological deficits, only pain in her nape which radiated to the left shoulder. Two days after the occipitocervical—C0–3 dorsal stabilization, we performed the MIS ASR surgery to remove the metastasis and free up the spinal cord. On the fourth day after the second surgery she was discharged to her home with relieved pain.

### Patient four

The male in his 40 s had a motorcycle accident, and the CT showed that he had suffered an Anderson-D’Alonso type II. Odontoid fracture, during which a 17 mm long cortical bone fragment from the process broke off and punctured the dura while causing compression to the medulla oblongata. The MRI scan confirmed liquor-leakage behind the odontoid process and showed injury to the posterior ligamentous complex. The patient was tetraplegic on admission. At the first step, we performed an emergency C1–2 fusion and the removal of the posterior arch of the C1 vertebra to decompress the medulla oblongata. Due to the patient’s instable circulatory system we could not perform the ventral decompressive surgery immediately, but only 7 days later. During this MIS ASR procedure, we removed the middle third of the odontoid process, as well as the haematoma and the cortical bone fragment causing the compression. In the course of the operation we also explored the dural injury, and successfully tamponaded and tissue-glued it. The patient is now self-sufficient.

## Discussion

The most common complications associated with transoral and transnasal odontoidectomy are CSF leakage, velopharyngeal insufficiency, wound dehiscence, pulmonary issues, meningitis and death [13]. In a systematic review, after analyzing 26 publications, Shriver et al. found that the only statistically significant difference between the complication rates of the two methods was the increased incidence of tracheostomy after transoral surgeries [13, 14]. Although the lateral routes provide a wider access, the risk of vertebral artery, jugular bulb and hypoglossal nerve injuries are higher [8–11]. Ponce-Gómez et al. found a significant difference between the time duration of the TO and EE approaches. TO surgeries lasted 141 min in average, while the EE-s lasted 238 min, p ≤ 0.02. Patients, who received the EE procedure, could be extubated immediately after the surgery, while patients who underwent the TO method stayed intubated for 24 h. Time until oral feeding was significantly shorter in the EE group, p ≤ 0.009 [15].

**Fig. 7:**
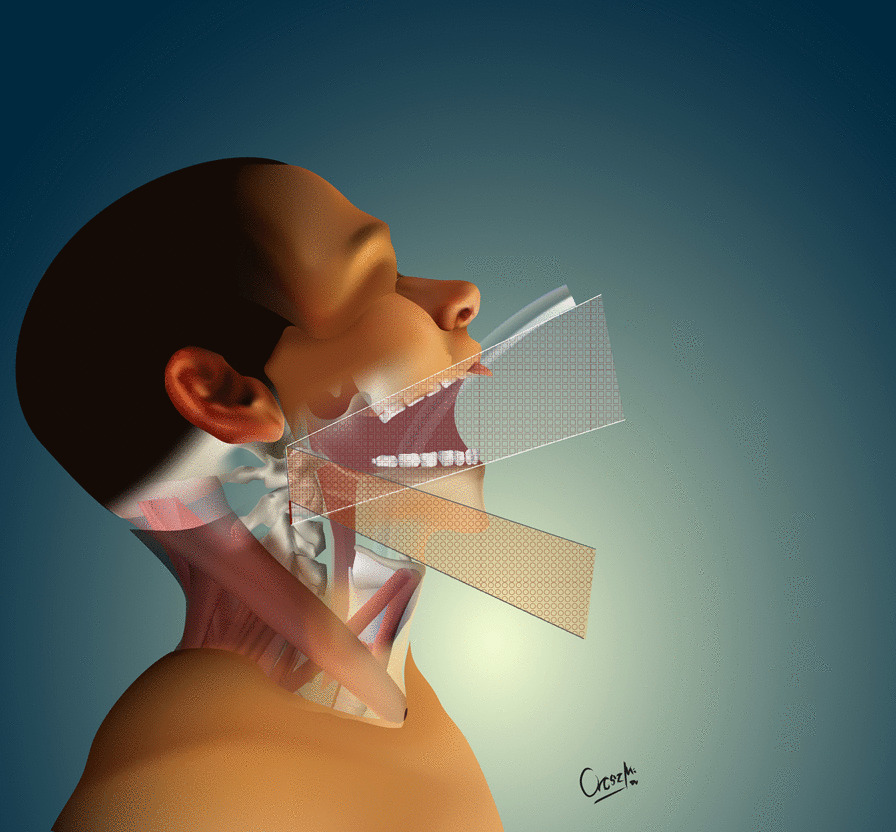
Schematic figure of different surgical interventions. Legend: white line: maximum decompression area with the transoral method, black line: maximum decompression area with the anterior submandibular retropharyngeal method, red line: the difference between the two methods’ decompression area

**Fig. 8:**
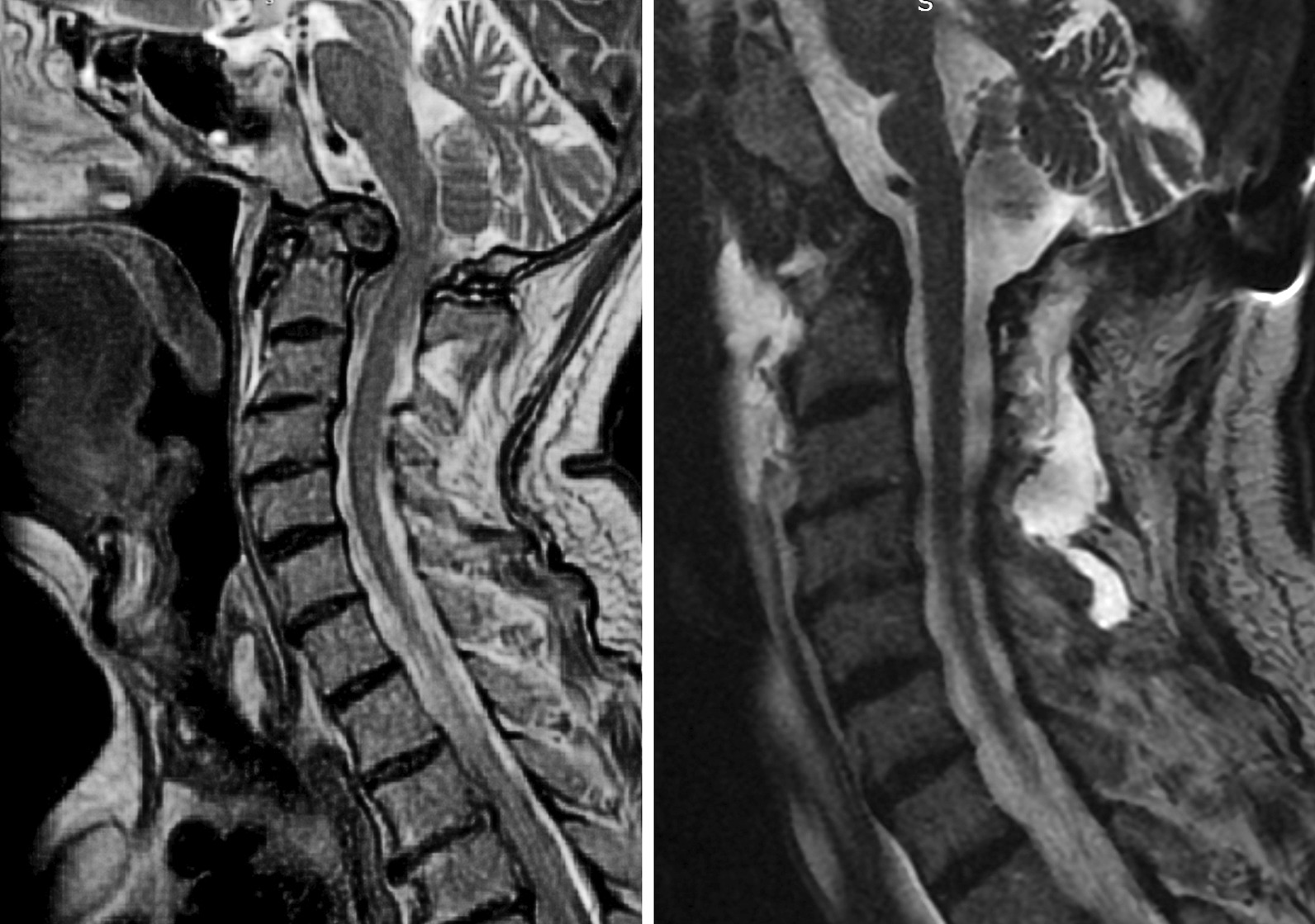
The preoperative (left) and postoperative (right) magnetic resonance (MRI-T2) imaging of the male patient in his 60 s. All images, figures and additional materials are the authors’, no materials are used from another source

The minimally invasive anterior submandibular retropharyngeal key-hole approach we used is a novel method for the decompression of C1, C2 ventral pathologies. From the four presented cases, we cannot come to long term conclusions, but we can state that during the MIS ASR—with the preservation of the hard and soft palates—the risk of velopharyngeal insufficiency associated with the TO method can be eliminated, as well as the risk of liquor-leakage. The blood-loss is minimal, the patients do not need tracheostomy, and can be started on oral diet within 24 h after surgery. The MIS ASR, compared to the TO method, provided on average 23% (4.56 cm^2^/6.05 cm^2^) smaller dural decompression area. We believe that this 1.40 cm^2^ difference occurs due to the fact that during the MIS ASR surgery—compared to the TO method—the distance between the C1 anterior tubercle and the midline of the corpus of C2 is seen at a more obtuse angle, reducing the possible area of decompression by 1.40 cm^2^ (Fig. 7). Nonetheless, the entire pathology could be removed and sufficient decompression could be achieved in all cases (Fig. 8). The small—25 mm wide -incision, the deep surgical field (94.3 mm on average), and the narrow surgical channel require proficient microsurgical skills.

## Conclusions

The novel, microscope assisted minimally invasive anterior submandibular retropharyngeal key-hole approach was feasible for dens resection in these four cases but further studies are required.

## Supplementary Information

Additional file1 (MP4 328267 KB)

## Data Availability

All data generated or analyzed during this study are included in this published article.

## Abbreviations

MIS ASR: Microscope assisted minimally invasive anterior submandibular retropharyngeal
TO: Transoral transpharyngeal odontoidectomy
EE: Endoscopic endonasal odontoidectomy
AR: Anterior retropharyngeal
MRI: Magnetic resonance imaging
CSF: Cerebrospinal fluid
CT: Computed tomography
